# East-Asian lupus nephritis in the Hopkins Lupus Cohort

**DOI:** 10.2478/rir-2023-0022

**Published:** 2023-09-27

**Authors:** Michelle Petri, Chenglong Fang, Daniel W. Goldman

**Affiliations:** Johns Hopkins University School of Medicine, Department of Medicine, Division of Rheumatology, Baltimore, Maryland, USA; Department of Rheumatology and Clinical Immunology, Peking Union Medical College Hospital, Chinese Academy of Medical Sciences& Peking Union Medical College; National Clinical Research Center for Dermatologic and Immunologic Diseases, Ministry of Science& Technology; State Key Laboratory of Complex Severe and Rare Diseases, Peking Union Medical College Hospital, Chinese Academy of Medical Sciences & Peking Union Medical College; Key Laboratory of Rheumatology and Clinical Immunology, Ministry of Education, Beijing 100730, China

**Keywords:** systemic lupus erythematosus, nephritis, epidemiology

## Abstract

**Background and Objective:**

East Asian systemic lupus erythematosus (SLE) is under represented in lupus cohorts outside of East Asia. We asked whether lupus nephritis was more common and more severe in East Asians than in other ethnicities in a large United States SLE cohort.

**Methods:**

The Hopkins Lupus Cohort, a longitudinal cohort of 2802 patients (53.5% Caucasian, 39.2% African-American, 3.2% East Asian) was studied. The SLICC/ACR Damage Index was used to assess renal outcomes. Results: East Asian patients had the same prevalence of lupus nephritis as African-Americans and both were higher than Caucasians. East Asians were not significantly different in frequency of end stage kidney disease compared with African-Americans. East Asians were more likely than Caucasians to have anti-Sm, low C3 and low C4. East Asians were more likely than African-Americans to have low C3 and low C4.

**Conclusion:**

East Asians living in the United States were more likely to have lupus nephritis than Caucasians. Poor outcomes such as end stage kidney disease occurred at an equal frequency in East Asians as in African-Americans. Lupus nephritis was both more frequent and more severe in East Asians than in African-Americans.

## Introduction

Lupus nephritis is one of the most severe manifestations of systemic lupus erythematosus (SLE). In the Hopkins Lupus Cohort, if lupus nephritis occurs in the first year, the risk of end stage kidney disease was 20% in 20 years.^[1]^ The current treatment of lupus nephritis is inadequate, with only about up to 40% complete renal response at one year, even with the addition of recently approved therapies such as belimumab^[2]^ and voclosporin.^[3]^ Many of the pivotal randomized clinical trials, including of mycophenolate mofetil^[4]^ of tacrolimus^[5,6]^ and of newer therapies such as low dose interleukin-2 (IL-2)^[7]^ have been done in East Asians. The complete renal response rates have overall been much higher in East Asians than in African-Americans, raising the question of whether East Asians have less severe lupus nephritis.

Multiple studies of the frequency of lupus nephritis have been done in East Asian countries.^[8,9]^ Comparisons of East Asian lupus nephritis with other races in the same cohort, however, have been quite limited. In the University of California San Francisco California Lupus Epidemiology Study (UCSF CLUES) study of 326 participants, lupus nephritis was more common in Asians (hazard ratio 2.9).^[10]^ In the Monash Lupus Clinic, lupus nephritis was more common (odds ratio 3.2) in Asians (58% of 51 patients than non-Asians (24%).^[11]^ We asked not just about the influence of East Asian race on lupus nephritis but also on end stage kidney disease in the Hopkins Lupus Cohort.

## Patients and Methods

The Hopkins Lupus Cohort has been approved by the Johns Hopkins University School of Medicine Institutional Review Board (Study number NA_00039294) on a yearly basis. Patients gave written informed consent. The cohort began in 1986; patients were seen at quarterly visits by protocol during which information on SLE activity, medications, and immunologic measures were recorded. The mean follow-up time was 16.8 ± 11.3 years (± Standard Deviation). All patients met American College of Rheumatology (ACR)^[12]^ and/or Systemic Lupus Erythematosus International Collaborating Clinics (SLICC) criteria^[13]^ for SLE.

Patients self-reported race by selection from the National Institutes of Health set of categories. Country of origin was established for patients who selected Asian as their race. The East Asian group consisted of those of East Asian or Southeast Asian ethnic origin, primarily China, Japan, South Korea, Taiwan, Philippines and Vietnam.

### Statistical analysis

A Chi-square test for categorical variables or the two-sample *t*-test for continuous variables (where appropriate) were used to determine whether there was a significant difference between characteristics of patients grouped according to race. The generalized linear model was used to adjust for age at last follow-up visit. All analyses were performed using JMP software, version 15.2 (SAS Institute, North Carolina, United States).

## Results

The analysis included 2802 patients from the Hopkins Lupus Cohort. The patients were 92% female, 53.5% (*n* = 1499) Caucasian, 39.2% (*n* = 1098) African-American, 3.2% (*n* = 89) Asian, and 4% (*n* = 116) other.

Table 1 shows the comparison of East Asians with Caucasians and African-Americans in terms of manifestations of lupus nephritis. East Asians were over 3-fold more likely than Caucasians to have lupus nephritis. East Asians had very similar lupus nephritis manifestations to African-Americans, with the exception that hematuria was significantly more frequent.

**Table 1:** Comparison of lupus nephritis manifestations between East Asians vs. Caucasians or African-Americans

Factor	East Asian (*n* = 89)	Caucasian (*n* = 1, 499)	OR (95% CI)	*P*-value	OR (95% CI) adj	*P*-value adj
Proteinuria	64%	35%	3.38 (2.16, 5.27)	<0.0001	3.12 (1.99, 4.89)	<0.0001
Nephrotic syndrome	30%	11%	3.30 (2.03, 5.37)	<0.0001	3.05 (1.87, 4.98)	<0.0001
Hematuria	46%	21%	3.11 (2.00, 4.82)	<0.0001	2.96 (1.90, 4.61)	<0.0001
Factor	East Asian (*n* = 89)	African-American (*n* = 1, 098)	OR (95% CI)	*P*-value	OR (95% CI) adj	*P*-value adj
Proteinuria	64%	58%	1.31 (0.83, 2.05)	0.2647	1.24 (0.79, 1.95)	0.3413
Nephrotic syndrome	30%	26%	1.20 (0.74, 1.93)	0.4513	1.11 (0.68, 1.80)	0.6801
Hematuria	46%	34%	1.67 (1.07, 2.59)	0.0259	1.61 (1.04, 2.50)	0.0346

Table 2 compares long-term outcomes. East Asians were over 2-fold more likely than Caucasians to develop end stage kidney disease.

**Table 2:** Comparison of lupus nephritis outcomes between East Asians vs. Caucasians or African-Americans

Factor	East Asian (*n* = 89)	Caucasian (*n* = 1, 499)	OR (95% CI)	*P*-value	OR (95% CI) adj	*P*-value adj
Renal insufficiency	31%	23%	1.49 (0.93, 2.37)	0.1188	1.92 (1.18, 3.13)	0.0082
Renal failure	10%	5%	2.05 (0.99, 4.25)	0.0549	1.95 (0.94, 4.05)	0.0738
Factor	East Asian (*n* = 89)	African-American (*n* = 1, 098)	OR (95% CI)	*P*-value	OR (95% CI) adj	*P*-value adj
Renal insufficiency	31%	36%	0.79 (0.49, 1.27)	0.3559	0.89 (0.55, 1.44)	0.6364
Renal failure	10%	12%	0.84 (0.41, 1.72)	0.7332	0.78 (0.38, 1.60)	0.4996

Table 3 examines whether a diference in prevalence of serologies might explain the higher risk in East Asians. East Asians were more likely than both Caucasians and African-Americans to have low complement. East Asians were more likely than Caucasians to have anti-Sm.

**Table 3:** Comparison of auto-antibody and complement in East Asians vs. Caucasians or African-Americans

Factor	East Asian (*n* = 89)	Caucasian (*n* = 1, 499)	OR (95% CI)	*P*-value	OR (95% CI) adj	*P*-value adj
Anti-dsDNA	70%	59%	1.63 (1.02, 2.60)	0.0432	1.52 (0.95, 2.44)	0.0817
Anti-Sm	36%	14%	3.57 (2.24, 5.68)	<0.0001	3.24 (2.02, 5.18)	<0.0001
Low C3	85%	50%	5.69 (3.13, 10.3)	<0.0001	5.38 (2.95, 9.79)	<0.0001
Low C4	66%	46%	2.30 (1.46, 3.61)	0.0003	2.09 (1.32, 3.29)	0.0016
Factor	East Asian (*n* = 89)	African-American (*n* = 1, 098)	OR (95% CI)	*P*-value	OR (95% CI) adj	*P*-value adj
Anti-dsDNA	70%	64%	1.34 (0.84, 2.16)	0.2466	1.30 (0.81, 2.09)	0.2817
Anti-Sm	36%	33%	1.13 (0.72, 1.79)	0.6351	1.03 (0.65, 1.64)	0.8987
Low C3	85%	57%	4.41 (2.42, 8.05)	<0.0001	4.19 (2.29, 7.66)	<0.0001
Low C4	66%	48%	2.07 (1.31, 3.27)	0.0018	1.95 (1.23, 3.09)	0.0044

**Figure 1:**
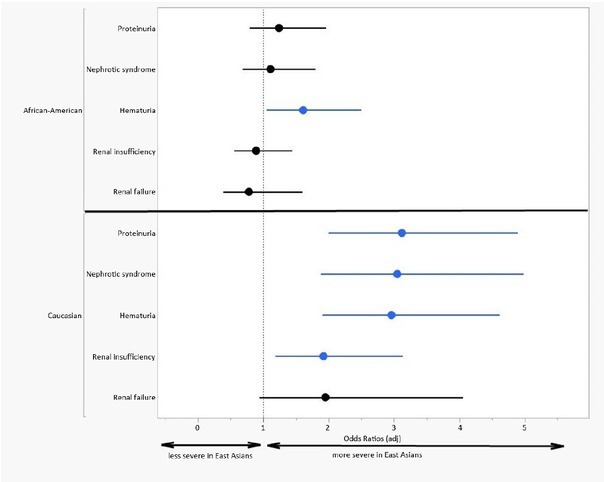
East Asian vs. African-American or Caucasian – Odds ratios for comparison of renal manifestations and outcomes.

The odds ratio for comparisons of major renal manifestations between East Asians and Caucasian or African-Americans is summarized in the forest plot shown in Figure 1.

**Figure 2:**
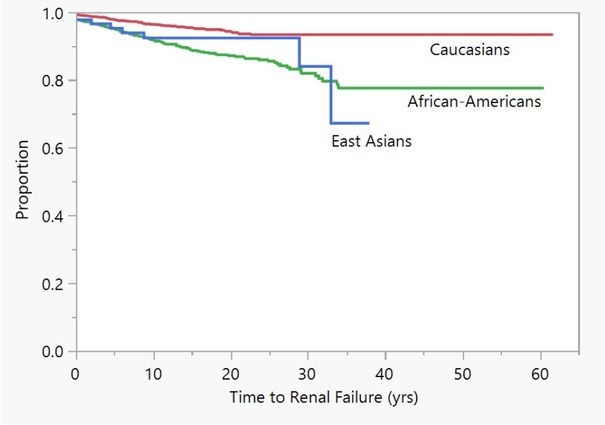
Time to end-stage renal disease in East Asians, Caucasians and African-Americans.

**Figure 3:**
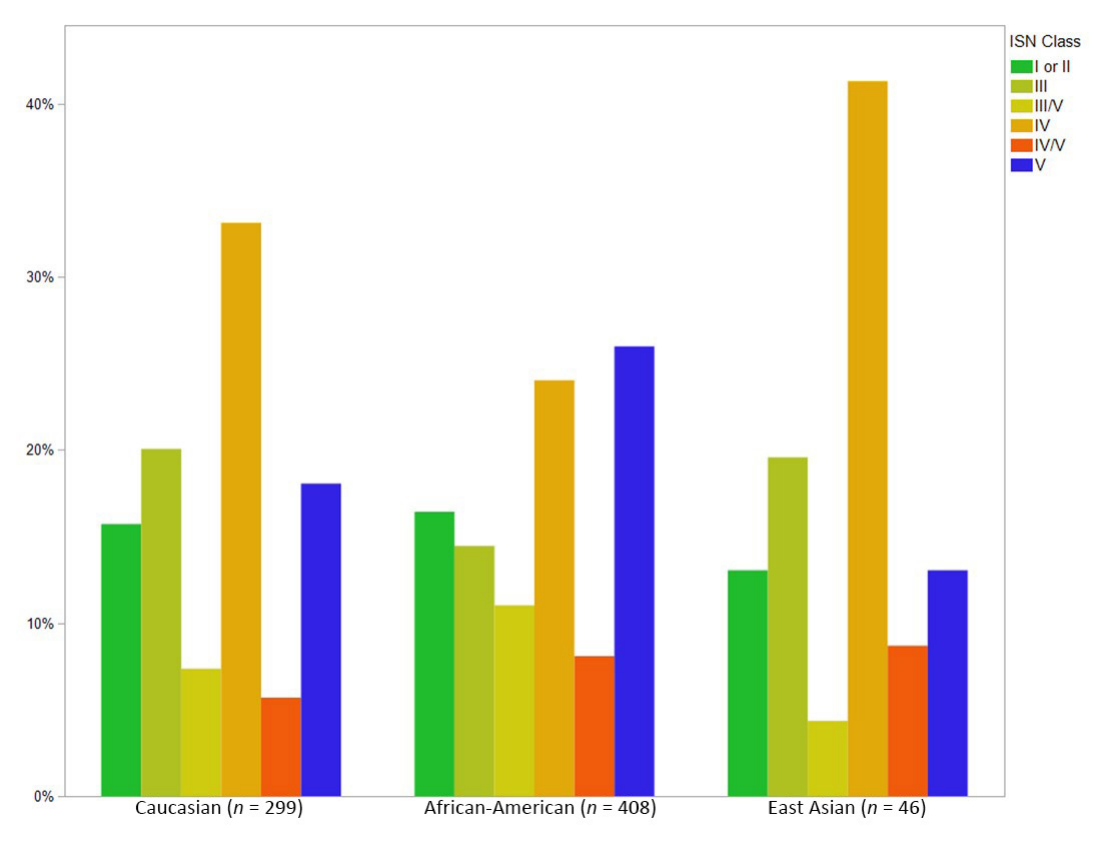
Distribution of lupus nephritis ISN Class at first biopsy in Caucasians, African-Americans and East Asians.

East Asians developed lupus nephritis earlier than Caucasians, but slower than African-Americans (Figure 2). Analysis of the first biopsy confirming lupus nephritis showed that East Asians were more likely to have proliferative lupus nephritis (ISN Class IV) and less likely to have membranous lupus nephritis (international society of nephrology (ISN) Class V) compared to Caucasians and African-Americans (*P* = 0.0082 Likelihood test) (Figure 3).

## Discussion

East Asian SLE patients in the Hopkins Lupus Cohort had the same prevalence of lupus nephritis as African-Americans and both African-Americans and East Asians had a higher prevalence than Caucasians (Table 1 and Figure 1). This fact, now proven in a third cohort [after the Australian experience^[11]^ and the UCSF experience^[10]^] should lead to greater respect for the risk of lupus nephritis in East Asians. Earlier studies highlighted only that East Asians had greater risk of lupus nephritis than non-Asians.

Long-term outcomes (Table 2) found equal frequencies of end stage kidney disease (Figure 2) between East Asians and African-Americans over. Earlier studies found that African-Americans and Hispanic Americans had more end stage kidney disease than Caucasians.^[14]^ Now, the severity in terms of East Asian lupus nephritis and end stage kidney disease has been proven.

Auto-antibodies and low complement are risk factors for lupus nephritis (Table 3). East Asians were more likely than Caucasians to have anti-Sm, low C3 and low C4. East Asians were also more likely than African-Americans to have low C3 and low C4. This may explain some of the excess risk in East Asians. Genome-wide association studies have also found different risk alleles for SLE in East Asians that may highlight other pathways.^[15]^

In terms of treatment, East Asians were significantly more likely than Caucasians or African-Americans to have used lupus nephritis treatments, including mycophenolate, rituximab and calcineurin inhibitors (Table 4). Thus, the worse outcome in East Asians did not reflect less intense therapy. Given that East Asians had the highest risk of Class IV diffuse proliferative glomerulonephritis, the worse outcome is expected.

**Table 4:** Comparison of treatment in East Asians vs. Caucasians or African-Americans

Factor	East Asian (*n* = 89)	Caucasian (*n* = 1, 499)	OR (95% CI)	*P*-value	OR (95% CI) adj	*P*-value adj
Mycophenolate use ever	53%	22%	4.07 (2.63, 6.31)	<0.0001	3.93 (2.54, 6.10)	<0.0001
Mycophenolate use current	35%	14%	3.33 (2.08, 5.31)	<0.0001	3.19 (1.99, 5.11)	<0.0001
Rituximab use ever	11%	4%	3.38 (1.66, 6.90)	0.0023	3.46 (1.68, 7.10)	0.0007
Calcineurin inhibitor use ever	17%	4%	4.47 (2.43, 8.21)	<0.0001	4.07 (2.20, 7, 51)	<0.0001
Methylprednisolone pulse ever	39%	29%	1.56 (1.00, 2.43)	0.0345	1.48 (0.95, 2.32)	0.0836
Factor	East Asian (*n* = 89)	African-American (*n* = 1, 098)	OR (95% CI)	*P*-value	OR (95% CI) adj	*P*-value adj
Mycophenolate use ever	53%	32%	2.40 (1.55, 3.72)	<0.0001	2.23 (1.43, 3.48)	0.0004
Mycophenolate use current	35%	22%	1.92 (1.21, 3.06)	0.0073	1.78 (1.11, 2.85)	0.0167
Rituximab use ever	11%	5%	2.36 (1.16, 4.79)	0.0262	2.19 (1.07, 4.48)	0.0310
Calcineurin inhibitor use ever	17%	7%	2.89 (1.58, 5.29)	0.0013	2.75 (1.50, 5.05)	0.0011
Methylprednisolone pulse ever	39%	39%	1.00 (0.64, 1.56)	NS	0.93 (0.59, 1.47)	NS

In conclusion, East Asians in the Hopkins Lupus Cohort had a similar frequency of lupus nephritis and of end stage kidney disease as African-Americans. East Asians had more high-risk serological tests. East Asians were more likely to have exposure to lupus nephritis treatments. These findings should increase awareness of the risks of lupus nephritis in East Asians in the United States.
